# A Multiscale Partition-Based Kolmogorov–Sinai Entropy for the Complexity Assessment of Heartbeat Dynamics

**DOI:** 10.3390/bioengineering9020080

**Published:** 2022-02-16

**Authors:** Andrea Scarciglia, Vincenzo Catrambone, Claudio Bonanno, Gaetano Valenza

**Affiliations:** 1 Bioengineering and Research Centre “E. Piaggio”, and Department of Information Engineering, School of Engineering, University of Pisa, 56122 Pisa, Italy; vincenzo.catrambone@ing.unipi.it (V.C.); g.valenza@ing.unipi.it (G.V.); 2 Department of Mathematics, University of Pisa, 56127 Pisa, Italy; claudio.bonanno@unipi.it

**Keywords:** signal processing, entropy, complexity, heart rate variability, cardiovascular dynamics

## Abstract

Background: Several methods have been proposed to estimate complexity in physiological time series observed at different time scales, with a particular focus on heart rate variability (HRV) series. In this frame, while several complexity quantifiers defined in the multiscale domain have already been investigated, the effectiveness of a multiscale Kolmogorov–Sinai (K-S) entropy has not been evaluated yet for the characterization of heartbeat dynamics. Methods: The use of the algorithmic information content, which is estimated through an effective compression algorithm, is investigated to quantify multiscale partition-based K-S entropy on publicly available experimental HRV series gathered from young and elderly subjects undergoing a visual elicitation task (Fantasia). Moreover, publicly available HRV series gathered from healthy subjects, as well as patients with atrial fibrillation and congestive heart failure in unstructured conditions have been analyzed as well. Results: Elderly people are associated with a lower HRV complexity and a more predictable cardiovascular dynamics, with significantly lower partition-based K-S entropy than the young adults. Major differences between these groups occur at partitions greater than six. In case of partition cardinality greater than 5, patients with congestive heart failure show a minimal predictability, while atrial fibrillation shows a higher variability, and hence complexity, which is actually reduced by the time coarse-graining procedure. Conclusions: The proposed multiscale partition-based K-S entropy is a viable tool to investigate complex cardiovascular dynamics in different physiopathological states.

## 1. Introduction

Measures of complexity such as Lyapunov exponents, entropies, and fractal dimensions may effectively characterize a nonlinear system through the analysis of its output observed as a time series [1]. In this context, under reasonable hypothesis of regularity, any time series can be considered as the image of a dynamical system under an observable function. Among the entropy quantifiers of dynamical systems such as the correlation integral [2], the approximate and sample entropies [3,4,5], the Kolmogorov–Sinai (K-S) entropy is a non-negative numerical value that takes into account the maximum amount of information needed to represent any time series generated by a dynamical system [6].

Previous studies suggest that complex dynamical systems should be investigated at different time scales: indeed, multiple scales might highlight different system behaviors, structures, and regularities, and the effects of external noise or perturbations can be reduced or limited [7,8]. To this extent, a multiscale approach of standard quantifiers such as sample and approximate entropy [7] has been introduced. Later, the multiscale analysis has been complemented with a multiscale extension of Lempel–Ziv compression [9], and other complexity estimates adopting techniques involving data compression [10] or not involving data compression [11]. Moreover, efforts have recently been devoted to reducing the parametric dependence of the entropy-like methods [12] even in a multiscale fashion [13]. Nevertheless, to our knowledge, a multiscale computation of K-S entropy has not been investigated yet for the study of physiological systems.

To this end, in this study a novel partition-based K-S entropy computation is proposed, which effectively extends K-S entropy to a multiscale domain. We thus define the multiscale Kolmogorov–Sinai Entropy (MKSE), whose values only depend on time scaling, along with the number of sets of the partitions: the method sensitively reduces the parametric dependence since the MKSE formulation does not depend on statistical parameters such as embedding dimension, tolerance, and the possibility of counting self-matches.

The proposed MKSE is here tested for the characterization of complex cardiovascular dynamics, represented though heart rate variability (HRV) series. Such series are obtained by the time intervals between two consecutive R-waves detected from the electrocardiogram, i.e., the R-R intervals. In the last decades, HRV studies using both linear and nonlinear modeling have been characterizing the influence of the autonomous nervous system (ANS) on the heartbeat. It is widely known, in fact, that cardiac dynamics follow a highly nonlinear and complex behavior [14,15,16,17,18] and hence they can be characterized in terms of complexity to provide relevant information on the underpinning physiological and pathological states [16,18]. Indeed, complexity is widely recognized as a useful biomarker of the health status of biological systems, being modulated by external stimuli, aging, and pathology [18].

The estimation of complexity may be linked to a symbolic analysis, and several approaches have been specifically applied to HRV series to this extent [11,19,20]. Briefly, symbolic analysis can be divided in two phases: first, the symbolization in which the original time series is translated into a new one by categorizing (assigning a symbol, hence a label, to) its numerical values, which generally leads to an information loss [21]. Symbolization may be performed by retaining the binary information of heartbeat series variation (i.e., an R-R interval decreasing or increasing with respect to the preceding one) [22], or by choosing the categories based on the distance from the series’ central value [23], or by assigning a symbol to each of the partitions in which the time series domain might be divided. The second phase regards the actual entropy estimation. In this frame, the alphabet entropy has been defined and exploited for the automatic classification of cardiac arrhythmias [21], while Shannon entropy was used to analyze cardiovascular regulation [11,22]. Moreover, a distribution entropy was proposed to study the differences between healthy aging and heart failure [24].

Focusing on multiscale approaches, quantifiers of multiscale entropy (MSE) have already been proposed for several applications. Exemplarily, MSE was exploited for the assessment of mood and emotional states [16], as well as to study the effects of orthostatic stress [25], diabetes mellitus [26], and aortic stenosis [27]. While MSE generally increases from low to high scales in healthy cardiac dynamics, MSE decreases in case of cardiac arrhythmia and congestive heart failure at specific time scales [7]. Regarding aging, previous studies suggested that complexity in heartbeat dynamics decreases with age [28,29].

Next, K-S entropy and its partition-based approach is introduced; then, methodological details of the proposed MKSE are reported, followed by the related experimental results on HRV series gathered from young and elderly subjects while watching the movie *Fantasia* [30], as well as from patients with congestive heart failure and atrial fibrillation [31,32].

### K-S Entropy

While deterministic systems are characterized by finite K-S entropy values, stochastic systems are associated with infinite K-S entropy values, and a positive or null K-S entropy characterizes chaotic and regular systems. The formal way to compute the K-S entropy of a time series consists of the following steps: identify the amplitude interval *I* of the series; divide *I* into a finite collection *Z* of disjoint sets (or, equivalently, defining a finite partition *Z* on *I*); compute the Kolmogorov–Sinai entropies relative to the finite partitions *Z*. Eventually, the K-S entropy is obtained by considering the supremum among all the partition-based K-S entropy values, which may vary throughout all possible finite partitions *Z* [6].

Thus, the K-S entropy associated with a specific partition quantifies the mean information needed to identify that specific sequence according to the chosen partition. With these assumptions, the behavior of a time series may be analyzed in terms of how predictable it is, which is related to the possibility of finding any repetitive patterns in the sequence of visited sets. It follows that the higher the predictability of a series, the lower its entropy value, since less information is required to describe repetitive patterns.

The first K-S entropy estimations have been proposed by Renyi [33], and Grassberger and Procaccia [3,34] with the generalized entropy of order 2, generally denoted by K${}_{2}$, which actually represents a lower bound of the K-S entropy. It has been used for its low computational cost but has the disadvantage of not recognizing cases where the system is chaotic or complex (i.e., K-S entropy is strictly positive) when K${}_{2}$ is null. The procedure of finding the supremum among all finite partitions for the K-S entropy computation can be avoided when the so-called *generating partitions* [35,36] are present. Such partitions are defined as the cases in which the supremum is actually reached. However, *generating partitions* are quite hard to be expressed in a closed form for a real time series, which generally are not linked to an analytical formulation: for this reason, it is best to set a finite partition on the amplitude interval *I* and compute the K-S entropy related to the chosen partition. As suggested in [37,38], the partition-based K-S entropy is also preferred to K-S entropy since it avoids situations where entropy is infinity, which is particularly useful in real-world systems perturbed by random noise.

To avoid numerical issues of the formal straightforward computation of the partition-based K-S entropy requiring vanishing measures of refined partitions, two tools taken from the field of information theory can be employed: the algorithmic information content (AIC) and the complexity *K* of symbolic strings. In more detail, symbolic strings correspond to infinite sequences ω whose elements are in a finite set *A* of symbols, named the alphabet. Intuitively, the AIC of a finite symbolic string σ can be described as follows: given a universal Turing machine (i.e., a generalization of the computer machine concept which executes any binary program), the AIC corresponds to the shortest length of any executable binary program giving the string σ as output. In other words, the binary program of minimum length which contains all the necessary instructions to reproduce the original series. The complexity *K* of an infinite symbolic string ω, instead, generalizes the concept of AIC for infinite symbolic sequences. The complexity *K* can be additionally extended to the orbits of dynamical systems (and time series) through symbolic dynamics, and, under suitable conditions, the complexity *K* happens to be equal to the partition-based K-S entropy. In other words, the K-S entropy relative to a partition is achieved through AIC and symbolic dynamics.

However, AIC and *K* are only theoretically achievable, since by definition there cannot be any real algorithm able to actually perform the AIC [39]. Practically, this issue can be overcome by using a lossless data compression algorithm, that is, a coding procedure allowing the reconstruction of an original symbolic series from its binary encoded representation. The binary encoded series contains all the instructions needed to reproduce the original one, consequently, the lossless data compression algorithms can approximate in practice what the notion of AIC represents in theory.

## 2. Materials and Methods

### 2.1. Experimental Setup and Data Processing

The experimental setup comprises two main parts: the first includes HRV series from the dataset *Fantasia* [29], publicly available at https://physionet.org/content/fantasia/1.0.0/, while the second includes data from the datasets *MIT-BIH* and *Congestive Heart Failure RR* [31,32], publicly available at https://physionet.org/content/nsrdb/1.0.0/, https://physionet.org/content/chf2db/1.0.0/, and https://physionet.org/content/afdb/1.0.0/, respectively (accessed on 31 January 2022).

The *Fantasia* dataset comprises electrophysiological signals collected from 40 subjects: 20 young (age range between 21 and 34 years old), and 20 elderly (age range between 68 and 85 years old) subjects. Each group comprised an equal number of men and women. All subjects provided a written informed consent and underwent a standard screening consisting in a physical examination, a routine blood count and biochemical analysis, an electrocardiogram (ECG), and an exercise tolerance test. Only healthy, nonsmoking subjects with normal exercise tolerance tests, and with no medical issues were admitted for the data collection. All subjects underwent 120 min of continuous supine resting state while the ECG was collected and digitized at 250 Hz. Subjects remained active while watching the movie *Fantasia* (Disney) to help maintain wakefulness [29]. Since some data showed the presence of artifacts, a total of 19 ECG recordings from the young and 18 recordings from the elderly groups were retained for further analyses. Starting from the ECG series, HRV series were derived through the identification of R-peaks using the well-known Pan–Tompkins algorithm [40]. HRV series were visually inspected for artifacts, which were eventually corrected through series preprocessed using *Kubios* software.

Moreover, unstructured, long-term cardiovascular recordings from 70 subjects were retained for further analyses. Specifically, 23 recordings were gathered from patients with atrial fibrillation (AF), with 10 h ECG series sampled at 250 Hz; 29 HRV series were gathered from patients (age range 34–79) with congestive heart failure (CHF) (NYHA classes I, II, and III), with 24 h Holter recordings sampled at 128 Hz; 18 long-term ECG recordings from healthy subjects (NS, age range 20–50), with ECG sampled at 128 Hz. HRV series from patients with AF and NS were derived from the ECG series through the well-known Pan–Tompkins algorithm for the identification of R-peaks. HRV series were visually inspected for artifacts, which were eventually corrected through series preprocessing by using *Kubios* software. A total of 63 recordings (29 CHF, 17 AF, and 17 NS) were retained for further analyses.

### 2.2. Kolmogorov–Sinai Entropy Related to a Partition

A dynamical system (X,μ,f) is a continuous map f:X→X with *X* a metric phase space where a probability measure μ is defined. In this study, it is required that *X* be a finite space and μ ergodic and preserved by the map *f*: in other words, heartbeat dynamics cannot be split into disjoint invariant subsets of *X*.

An orbit $\left(x_{0},x_{1},\ldots ,x_{n},\ldots \right)$ with $x_{i}=f^{i}\left(x_{0}\right)$ for i>0 is a sequence of points that describes the temporal evolution of the dynamical system starting from the initial condition $x_{0}$. In order to identify the dynamics, it can be convenient to divide the space *X* into a finite collection of disjoint sets $Z={\left\{I_{i}\right\}}_{i=1...N}$: this space division is called measurable finite partition if all the sets $I_{i}$ are measurable, their union is equal to *X*, and all the pairwise intersections form sets whose μ-measure is 0 [39].

A finite partition *Z* defines the quantity

(1) $$H_{\mu }\left(Z\right)=-\sum_{i=1}^{N}\mu \left(I_{i}\right)\log\left(\mu \left(I_{i}\right)\right),$$

known in the literature as the Shannon entropy, which returns a numerical value referred to the amount of information needed to describe the position of a point in the phase space *X* according to the measure μ and the partition *Z*. From the Shannon entropy, a notion of complexity of the orbit, and hence of the dynamical system, has been defined. It starts by noting that the set $Z_{n}$, formed by all the intersections of the pre-images $f^{-j}\left(I_{i}\right)$ for all i=1,…N and for j=0,…,N−1, is still a finite partition and any of its elements describes an orbit sequence of length *N* according to the partition elements visited at each time by the dynamical system. The Kolmogorov–Sinai entropy $h_{\mu }\left(f,Z\right)$ of a dynamical system is thus defined as

(2) $$h_{\mu }\left(f,Z\right)=\lim_{n\to \infty }\frac{1}{n}H_{\mu }\left(Z_{n}\right)$$

and it measures the average amount of information required to identify any point of a generic orbit of a dynamical system *f* [39]. The lower this entropy is, the less complex and more predictable the output of the system.

### 2.3. AIC and Complexity

In this study, to estimate the partition-based Kolmogov-Sinai entropy, the approach based on the AIC and, in general, on the complexity *K* of a symbolic string was chosen.

First of all, let A={1,…,N} be the finite alphabet composed by a total of *N* symbols: the sequences with points in the set *A* are called symbolic strings. Let Ω denote the space of all symbolic strings with infinite length and whose elements belong to the same finite alphabet *A*, and let Σ be the set of all sequences with elements in *A* of length *m* [39].

A quantifier of the behavior of any string σ∈Σ can be expressed through the AIC. Briefly, let *C* define a computer which takes a binary string *P*, defined as the program, as an input and that returns after some computations a string σ=C(P) whose elements belong to some alphabet *A*. The AIC of a string σ, relative to the computer *C*, is defined as the shortest binary program *P* that returns σ as its output, namely AIC(σ,C)=min{|P|:C(P)=σ}. The program *P* has all the minimal nonredundant information to describe the points of the string σ and hence, it is considered a sort of string encoding. It follows that the more occurrences of similar patterns are in the series, the less quantity of information is required to characterize the series [39]. Actually, computer *C* has to be *universal*, that is, it can simulate any other computing machine. Since the asymptotic behaviors of AIC(σ,C) and $AIC(\sigma ,C^{\prime })$ do not differ for different universal computing machines *C* and $C^{\prime }$ [41], AIC(σ) can be considered instead of AIC(σ,C). From AIC, it is thus possible to define the algorithmic complexity *K* for infinite strings ω∈Ω as

(3) $$K\left(\omega \right)=\underset{n\to \infty }{lim sup}\frac{AIC(\omega^{n})}{n},$$

where $\omega^{n}$ is the string ω truncated at the *n*th element. Note that the complexity *K* formally corresponds to the shortest binary program length that, on average, returns any element of the symbolic string when executed by a universal computing machine. Practically, this program length is proportional to the minimal information needed, on average, to specify any of its element [39]. As before, lower values of complexity *K* are associated with more predictable symbolic strings.

### 2.4. Symbolic Dynamics

The complexity *K* of infinite strings and the partition-based Kolmogorov–Sinai entropy for infinite orbits are strictly related.

For a dynamical system (X,μ,f) on the finite metric space *X* with a finite partition $Z=\{I_{1},\ldots ,I_{n}\}$, where *n* represents the cardinality of the partition, such relation is guaranteed through the symbolic dynamics, or rather a bijection ϕ:X→Ω which converts any orbit with an initial point x∈X into a symbolic string $\omega =(\omega_{0},\omega_{1},\omega_{2},\ldots )$ according to the following rule: $\omega_{i}=k\Leftrightarrow f^{i}\left(x\right)\in I_{k}.$ In other words, the procedure is assigning for each point of the orbit a specific symbol associated with the set of the partition it is visiting.

Through the identification above, it is also possible to extend the definition of complexity *K* to any orbit of a dynamical system with a partition *Z*: for any orbit $\left(x_{0},x_{1},x_{2},\ldots \right)$ with initial condition $x_{0}$ the complexity $K(x_{0},Z)$ corresponds to the complexity K(ω) where $\omega =\phi (x_{0})$ is the associated symbolic string. In the significant case of ergodicity, Brudno [42] showed that:

(4) $$K\left(x,Z\right)=h_{\mu }\left(f,Z\right)$$

for μ-almost any x∈X and for all finite measurable partitions *Z* of *X*. Moreover, under the same hypothesis of Equation (4), the following relation also holds [43,44]

(5) $$\underset{n\to \infty }{lim inf}\frac{AIC(\omega^{n})}{n}=h_{\mu }\left(f,Z\right)$$

for μ-almost any x∈X and for all finite measurable partitions *Z* of *X*, which guarantees that, asymptotically and except for a set of 0 μ-measure, the mean algorithmic information content of any string cannot achieve lower values than the partition-based K-S entropy. In particular, by jointly considering Equations (4) and (5), the computation of the K-S entropy for a partition $h_{\mu }\left(f,Z\right)$ is achieved in the limit by the algorithmic complexity K(x,Z) and, consequently, the AIC, without requiring the limsup procedure of the straightforward formal definition.

The entropy estimation is thus reduced to the evaluation of the AIC. As mentioned before, this quantity is only theoretically achievable since the AIC is not computable, or rather there does not exist an algorithm able to perform it. This is an equivalent statement of Turing’s halting problem [45,46] and can also be associated with a strong version of Gödel’s incompleteness theorem, which is expressed through the framework of computational complexity theory [47]. Consequently, the AIC is approximated through lossless data compression algorithms. The compressed string, as the binary program of the AIC, contains all the instructions to identify the original string: the shorter the length of the encoded string, the more efficient the AIC approximation. Among the available lossless data compression algorithms, in the present study the CASToRe compression algorithm was chosen [48], due to its speed of convergence, i.e., the characteristic of generating binary encoded messages of extreme short lengths. Its formulation has been effective for the study of weakly chaotic dynamical systems [48].

Notably, computing the AIC and hence the partition-based K-S entropy without CASToRe algorithms entails choosing statistical parameters such as embedding dimension and tolerance, and it involves the possibility of counting self-matches, whose computation is not always straightforward and often requires interpretation [49].

### 2.5. Multiscale Analysis

Given a one-dimensional time series $\left\{x_{1},\ldots ,x_{N}\right\}$, coarse-grained time series $y^{\left(\tau \right)}$ are constructed according to the scale factor τ. The construction is as follows [50]: the original series is divided into non overlapping windows of length τ and then an average is computed among all the points of each window. In symbols, the generic element of the coarse-grained time series is given by the equation

(6) $$y_{j}^{\left(\tau \right)}=\left(1/\tau \right)\sum_{i=(j-1)\tau +1}^{j\tau }x_{j},\;1\leq j\leq N/\tau .$$

Substantially, if τ=1 then $y_{j}^{\left(1\right)}$ is exactly $x_{j}$ for all j∈{1,…,N}. It is immediate that the length of each coarse-grained series $y^{\left(\tau \right)}$ is equal to the length of the original series, *N*, divided by the scale factor τ.

Summarizing, for each subseries, the partition-based K-S entropy is computed and plotted as a function of the scale factor τ, thus defining a multiscale K-S entropy (MKSE). The proposed methodology extends to the multiscale domain with a preliminary analysis reported in [51].

### 2.6. Complexity Estimation Procedure

Here the complexity estimation procedure for the two experimental setups is described. In the first setup, comprising series from elderly and young subjects, HRV series were interpolated at a sampling frequency of 2 Hz to obtain series of equal length, thus avoiding possible confounding factors; then subsequences were extracted from the interpolated ones according to Equation (6). Each subsequence was converted into a symbolic one with respect to a uniform partition. The use of uniform partitions, i.e., formed by a finite number of disjoint sets of the same length, simplifies the numerical computation and allows for consistent results as the number of sets of the partition increases [36]. Then, the CASToRe algorithm was applied to compute the complexity $K^{\left(\tau \right)}\left(x,Z\right)$, thus obtaining the K-S entropy $h_{\mu }^{\left(\tau \right)}\left(f,Z\right)$, where τ denotes the scaling factor of the subsequence.

More specifically, the interval I=[0.60,1.84] s was obtained as the subject-wise range of heartbeat series, independently on subjects and time scale: *I* represents the complete range between the minimum and maximum interbeat interval across all subjects and factor time scaling. *I* was divided by a cardinality of partitions *n* going from 2 to 20 (i.e., a total of 19 different partitions). The scaling time τ was chosen in the range (1,…,10), thus considering series with at least 1000 points as basic practice for data compression.

Finally, series were converted to strings, and the CASToRe algorithm was applied to compute the K-S entropy [48] (Equations (3) and (4)) for each time scale τ and cardinality of the partition *n* considered.

In the second experimental setup, instead, each HRV series was truncated at 10,000 samples in order to avoid biases due to series length, since they represent long-term cardiac activities. Then, the complexity estimation followed the procedure described above, with the exception of considering an interval I=[0.32,1.25] s (the inferior limit was due to arrhythmic phases).

### 2.7. Statistical Analysis

In the first experimental setup (Fantasia), MKSE values at each scale and partition obtained from artifact-free HRV series for the two groups (i.e., elderly and young) were compared through nonparametric Mann–Whitney tests for unpaired samples, with the null hypothesis of equal median between the populations. The uncorrected statistical significance was set to $\alpha_{0}=0.05$, and a threshold of α was defined in accordance to the Bonferroni rule of correction for multiple comparisons, considering the number of scales for a fixed partition (i.e., $\alpha =\frac{\alpha_{0}}{10}=0.005$).

In the second setup, multiscale partition-based K-S entropy estimations at each partition and scale for NS, CHF, and AF groups were statistically compared through the nonparametric Kruskal–Wallis test, with the null hypothesis of equal median between the populations. The corrected statistical significance α was set to 0.005 (see above), and eventual pairwise multiple comparisons were performed through the Mann–Whitney test, with a corrected significance threshold set to $\frac{\alpha }{\#\mathrm{comparisons}}=\frac{0.005}{3}\approx 0.0017$.

**Figure 1:**
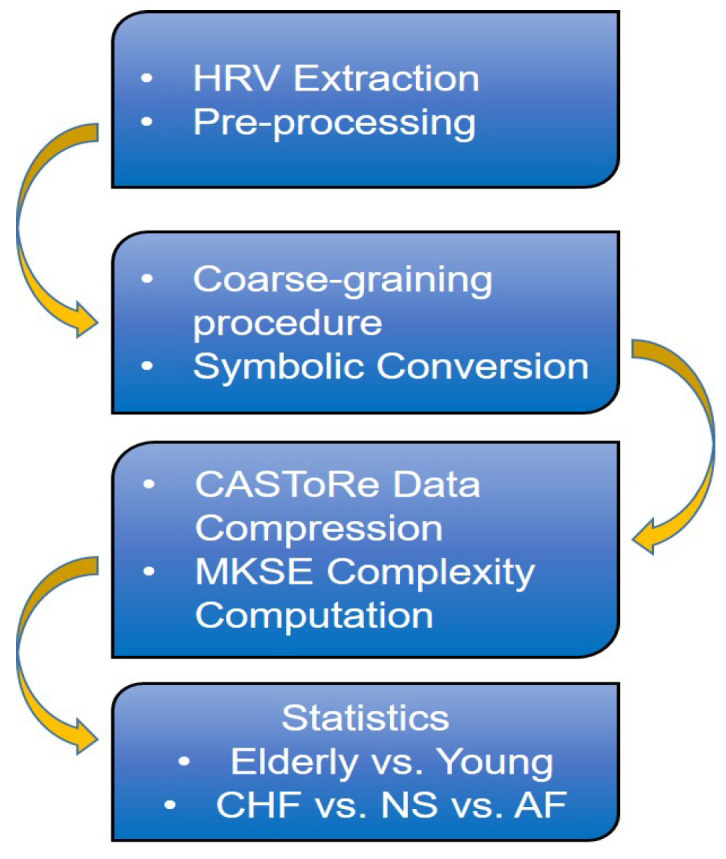
Block scheme of the experimental procedure. HRV series are derived from ECG series and are eventually preprocessed to correct artifacts. Then, artifact-free series are coarse-grained according to a multiscale approach and converted into symbolic series. The lossless data compression CASToRe is then applied to compute K-S entropy at each partition and scaling time, for which group-wise inferential statistics are finally performed to evaluate the elderly vs. young and CHF vs. NS vs. AF differences.

Figure 1 shows the overall block scheme of the experimental procedure.

## 3. Results

### 3.1. Experimental Setup 1: Elderly vs. Young Subjects (Fantasia)

**Figure 2:**
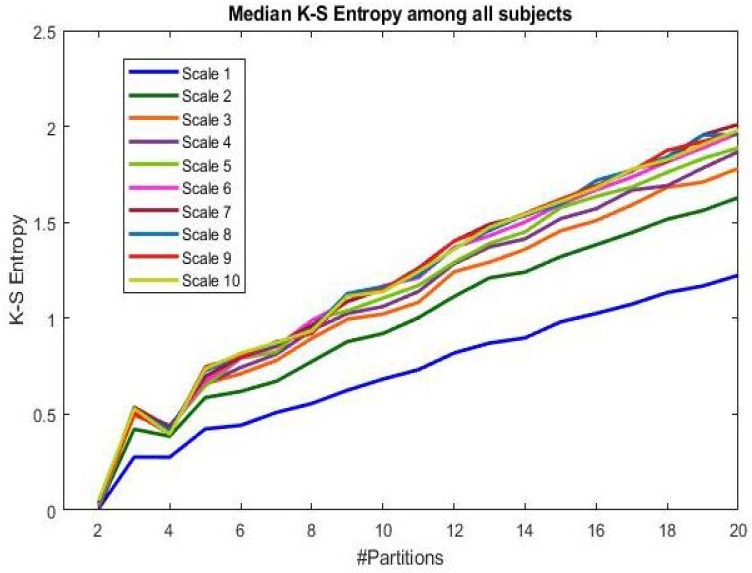
Median across all subjects (i.e., both young and elderly groups) of the MKSE as a function of the cardinality of the uniform partitions considered.

Figure 2 shows the behavior of the MKSE as a function of the cardinality of the partition. Note that in this case, no differentiation has been made among the subjects’ groups to appreciate how the MKSE behaves with respect to partition cardinality *n* and the time scaling τ, independently from the subjects’ aging factor. Each line corresponds to the median entropy value among all the subjects’ series at the time scaling τ considered, from one to ten. Generally, the higher the time scaling and cardinality of the partitions, the higher the MKSE. At each scale, the MKSE value tends to decrease for partitions with two or three sets, and then increases for partitions of cardinality greater than four. Looking at the differences in terms of time scaling, it seems that the MKSE values increase at higher scales for all partitions.

**Figure 3:**
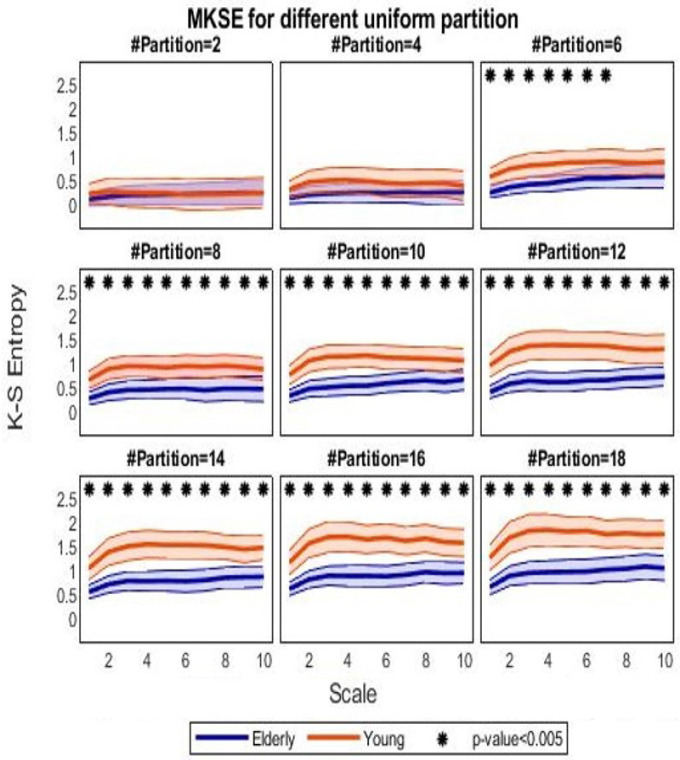
MKSE values for the two groups. Orange lines refer to MKSE values extracted in the young groups, whereas blue lines represent MKSE for the elderly ones. Thick lines indicate the median across subjects, whereas chromatic shaded areas represent the median absolute deviations. Each subfigure reports MKSE values (vertical axis) as a function of the time scaling τ (horizontal axis) for a different partition cardinality (even), increasing in the left-right and top-down directions from 2 to 18. Asterisks indicate corrected *p*-value <α=0.005 from a nonparametric Mann–Whitney test for unpaired samples.

Figure 3 shows the MKSE group-wise trends as a function of the ten different time lags τ for different uniform partitions, at even cardinality spanning from 2 to 18. At a first glance, it can be observed that MKSE values are higher with HRV series from the young cohort compared to the ones associated with the elderly, for almost every uniform partition; nonetheless, the MKSE values are almost indistinguishable when partitions are too coarse, i.e., with cardinality up to four in this case. Figure 3 shows that the finer the partition, the higher the MKSE values, independently of scales. MKSE generally increases from no-scaling to τ equal to 4. Moreover, it seems that MKSE for the young presents a slightly higher deviation, which was calculated through the median absolute deviation, with respect to the elderly.

**Figure 4:**
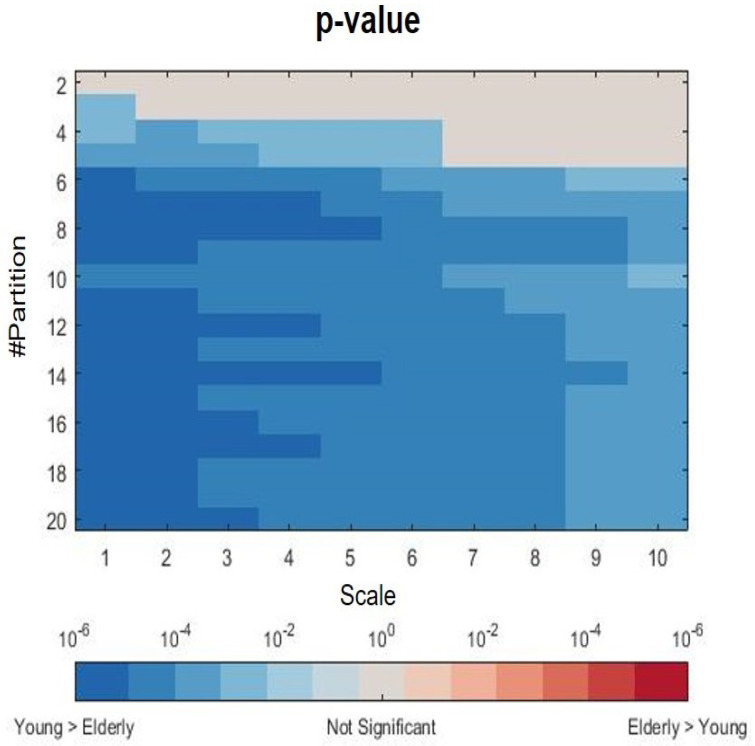
Distribution of *p*-values from nonparametric Mann–Whitney test for unpaired samples for the young vs. elderly comparison for each scale and partition.

The coarse-graining procedure tends to regularize time series; the median lines are closer at higher time scale. In particular, statistical significance tends to increase with the cardinality of the partitions and tends to decrease with the scaling time. Figure 4 shows the *p*-values from the nonparametric Mann–Whitney test for unpaired samples evaluated at each partition and scale, with a minimum value (in the darkest areas) as low as approximately $10^{-6}$.

### 3.2. Experimental Setup 2: NS vs. CHF vs. AF

**Figure 5:**
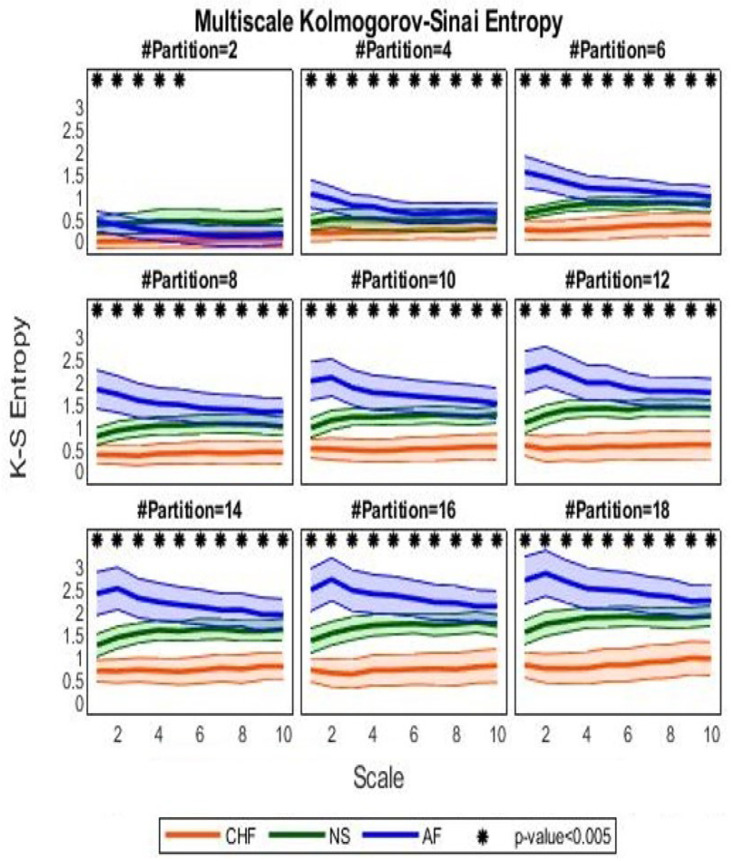
Median MKSE values as a function of scaling time. Orange lines refer to MKSE values extracted in the CHF group, green lines represent MKSE for healthy controls, and blue lines stand for the MKSE of arrhythmic HRV series. Thick lines indicate the median across subjects, whereas chromatic shaded areas represent the median absolute deviations. Each subfigure reports MKSE values (vertical axis) as a function of the time scaling τ (horizontal axis) for a different partition cardinality (even), increasing in the left-right and top-down directions from 2 to 18. Asterisks indicate the corrected *p*-value <α=0.005 from a nonparametric group-wise Kruskal–Wallis test.

Figure 5 shows the MKSE trends as a function of 10 different scaling times τ for different uniform partitions, whose cardinality spans evenly from 2 to 18. It can be observed that higher median MKSE values are associated with AF at any scaling time but for a two-element partition, while lower median MKSE values are associated with the CHF group. Moreover, AF estimates are characterized by a high dispersion (median absolute deviation) around the median. Conversely, the NS group is associated with MKSE values less dispersed around the median trend. Interestingly, it is also observed that MKSE is monotonically increasing independently on the three groups when it is regarded as a function of partition cardinality; on the other hand, if MKSE is seen as a function of the scale factor τ, the quantifier appears decreasing for the AF cohort and increasing for the remaining series (i.e., NS and CHF) until a plateau is reached.

A statistical difference between NS and CHF can be observed at partitions with cardinality greater than 6, at any scale; AF and CHF patients are statistically different at partitions of cardinality greater than 4, while AF and NS show differences at small time delays τ.

**Figure 6:**
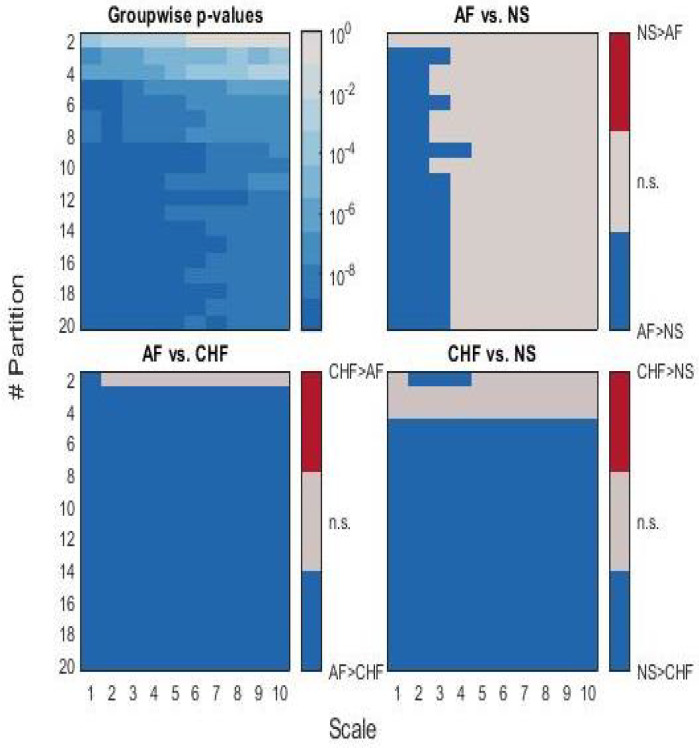
Graphical representation of the statistical analysis results. Top-left corner: *p*-values from a nonparametric Kruskal–Wallis test (i.e., AF vs. CHF vs. NS) for each scale and partition. The other images represent results of pairwise comparisons through non-parametric Mann–Whitney test for unpaired samples: light grey areas represent non-significant differences in terms of MKSE with *p*-value $>\alpha_{1}=0.00167$, whereas blue areas denote *p*-value $<\alpha_{1}$.

Figure 6 shows the quantitative results from the nonparametric statistical analysis.

## 4. Discussion

In this study, a novel approach for the estimation of a multiscale Kolmogorov–Sinai entropy was proposed and evaluated on publicly available HRV datasets. In a first experimental setup, HRV series gathered from 19 young and 18 elderly subjects while watching the movie *Fantasia* [29] were analyzed; in a second setup, 17 HRV time series gathered from healthy subjects, 17 HRV time series from arrhythmic subjects, and 29 from patients with CHF were analyzed [31,32].

The MSKE is a complexity quantifier aiming to numerically evaluate scale-dependent structures in a time series. As partition-independent K-S entropy may be characterized by an infinite value when estimated on noisy or perturbed dynamical systems, such as the cardiovascular system, in this study, the MKSE was estimated as a multiscale expansion of the partition-based Kolmogorov–Sinai entropy, which always returns finite values [37]. To overcome numerical and theoretical issues of the straightforward computation, such as the lack of analytic expressions for an ergodic dynamical time system (i.e., cardiovascular system), the partition-based K-S entropy was here evaluated by converting the time signals into symbolic series, and then approximating the AIC through the CASToRe lossless data compression algorithm [48]. This approach provides a reliable estimation while avoiding the choices and hard evaluations of statistical parameters, such as embedding dimension, tolerance, or the possibility of counting self-matches.

The results demonstrated that the proposed MKSE is a viable tool to discern among the different stages of psychophysiological and cardiovascular conditions. Specifically, statistically significant differences were found between young and elderly subjects, as well as between healthy and dysfunctional cardiovascular dynamics. Hence, MKSE could profitably be used as a biomarker quantifying complexity in heartbeat series. At each time scale, the K-S entropy values tended to increase as the cardinality of the partitions grew. This is mainly due to the fact that the considered time series had a finite length: the higher the number of intervals, the more symbols are needed to represent them, and consequently, there are fewer occurrences of similar or predominant patterns in the associate symbolic finite series [39].

Furthermore, when MKSE was considered as a function of time scales, independently from the partitions, it was confirmed that MKSE, as a proposed multiscale measure, reached a maximum and then slightly decreased as time scaling increased [7]. This should be a consequence of the regularity effect given by the extraction of subseries from the original one (see Equation (6)).

Looking at the results from the Fantasia dataset, it was also confirmed that heartbeat complexity level was significantly higher in young adults than in elderly ones: the difference between the two groups was statistically significant at each scale when a partition with more than six ranges was taken into account. From an information theoretic viewpoint, the lower values of partition-based MKSE extracted from series belonging to the elderly subjects suggest that a lower amount of information is required to describe the associated heartbeat dynamics with respect to the series of young participants; equivalently, the converted symbolic HRV series in the elderly subjects shows more repetitive patterns when compared to series from the other group. To be noted, the prominent dispersion of the median MKSE for the young population reflects the unpredictability of the converted symbolic series. It was found that a uniform partition with cardinality at least equal to six was needed to significantly discern the two groups. This might be due to the fact that when the partition is too coarse (i.e., composed by few intervals), more HRV values fall in the same interval and consequently have the same string. Conversely, a finer partition allows a more diversified HRV series (e.g., a series from the young group) to span the entire alphabet of the symbolic conversion with respect to a stationary HRV series (e.g., a series from the elderly group). This results in a higher possibility to differentiate among the two groups at finer partitions, with respect to coarser ones.

Regarding the results from the second experimental setup, the three groups (i.e., CHF, AF, and NS) showed different MKSE behaviors at different scales and cardinalities of partition. While AF was associated with higher MKSE values than NS, the NS series were more complex than the CHF ones. Such a difference was statistically significant when the complexity estimation was performed with partitions of cardinality greater than 5 along with a scale not exceeding 3.

Subseries extraction procedure for the multiscale approach tended to decrease the complexity of AF dynamics: for this reason, pairwise significant differences were not jointly achieved for scales higher than 3. This phenomenon was emphasized for partition with two sets, where AF series seemed less complex than NS ones. Moreover, since a higher complexity stands for more unpredictability, the actual unpredictability of AF series was highlighted not only by their median MKSE values, but also by their dispersion around the median. Unlike other quantifiers [7], we remark that the proposed method was able to statistically discern NS and CHF groups at time delay τ=1 for almost any partition (here, only partitions with cardinality at most 4 were excluded).

## 5. Conclusions

The proposed MKSE method, using the AIC approximation with the CASToRe lossless data compression algorithm, was able to statistically discern among different psychophysiological and pathological states of cardiac dynamics. The MKSE tended to remain stable at different time scales and partitions, where other methods showed more variations [9].

Our findings are in agreement with previous studies suggesting that aging and CHD reduce the complexity of the heartbeat series [7,28,30,52,53] The proposed method does not require parameter tuning such as embedding dimension, tolerances or the possibility of counting self-matches [54], whose computation may be challenging [49]. Therefore, the MKSE may be used as a potential biomarker for heartbeat dynamics analysis, complementing state-of-the-art methods for cardiac complexity assessment.

As a future development, the MSKE may be evaluated on large datasets for the analysis of different pathophysiological conditions, especially in the case of neural and/or cardiovascular disorders.

## Data Availability

Data are publicly available at: https://physionet.org/content/fantasia/1.0.0/; https://physionet.org/content/nsrdb/1.0.0/; https://physionet.org/content/chf2db/1.0.0/ and https://physionet.org/content/afdb/1.0.0/ (All accessed on 31 January 2022).
